# Neuropathy in Human Immunodeficiency Virus: A Review of the Underlying Pathogenesis and Treatment

**DOI:** 10.7759/cureus.25905

**Published:** 2022-06-13

**Authors:** Lakshya Motwani, Nailah Asif, Apurva Patel, Deepanjali Vedantam, Devyani S Poman

**Affiliations:** 1 Research, Smt. Nathiba Hargovandas Lakhmichand (NHL) Municipal Medical College, Ahmedabad, IND; 2 Research, Ras Al Khaimah (RAK) College of Medical Sciences, Ras Al Khaimah, ARE; 3 Research, Gujarat Medical Education & Research Society (GMERS) Gotri Medical College, Vadodara, IND; 4 Internal Medicine, Kamineni Academy of Medical Sciences & Research Center, Hyderabad, IND; 5 Research, Smolensk State Medical University, Smolensk, RUS

**Keywords:** distal symmetrical polyneuropathy, peripheral sensory neuropathy, highly active antiretroviral treatment, acquired immune deficiency syndrome (aids), chronic inflammatory demyelinating neuropathy, peripheral neuropathy, hiv aids

## Abstract

This article explores the various causes of the human immunodeficiency virus (HIV), and its associated neuropathy, including the effects of HIV on the nervous system and the long-standing therapy that is often provided to patients with HIV. Several studies regarding the neurotoxic effects of combined antiretroviral therapy (cART) and HIV were reviewed and various hypotheses were discussed. Furthermore, we present the nature of HIV-sensory neuropathy (HIV-SN) among different demographic populations and their subsequent risk factors predisposing them to this condition. It was observed that the incidence of the disease increases in increased survival of the patients as well as in males. Finally, the current approach to HIV-SN and its overlapping features with other causes of peripheral neuropathy have been discussed which demonstrates that a clinical examination is the most important clue for a healthcare professional to suspect the disease. Our main aim was to study the current perspectives and guidelines for diagnosing and managing a patient with HIV-SN to reduce disease prevalence and bring about a more aware frame of mind when following up with an HIV patient.

## Introduction and background

Acquired immune deficiency syndrome (AIDS) is a disorder characterized by gradual loss of cell-mediated immunity due to an infection by the human immunodeficiency virus (HIV). The AIDS epidemic hit the world when the first case was discovered in 1981. It was deemed incurable before the advent of highly active antiretroviral therapy (HAART). According to the Joint United Nations Programme for HIV/AIDS (UNAIDS), as of 2016, HIV/AIDS affects roughly 36.7 million people around the world [1]. The UNAIDS has also stated that cohorts containing sex workers and their clients, men who have sex with men (MSM), homosexuals, transgender individuals, and injection drug users are at the highest risk for contracting the disease. The incidence of new infections has reduced among males but remains relatively stable among the female population [2]. This disease presents insidiously with opportunistic infections and a near-decade-long latent period. The human immunodeficiency virus attacks the immune system and has a special predilection for the helper T (thymus) lymphocytes. Currently, the Center for Disease Control (CDC) guidelines state that the screening test for HIV/AIDS should include a fourth-generation test for detecting the presence of both the p24 antigen and antibodies formed against it [3]. To monitor therapy and the general progress of the disease, CD4+ T cell count is considered superior as compared to HIV viral load. Presently, HIV-AIDS is managed with lifelong HAART, and people living with HIV (PLHIV) have made it part of their lifestyle. Highly active antiretroviral therapy includes combinations of antiviral drugs that prevent active HIV ribonucleic acid (RNA) replication. Palliative therapy to reduce the oncoming infections, safe practices like protection, and maintenance of beneficial attitudes are also of utmost importance to keep the disease at bay.

Nearly 40% of these patients suffering from HIV develop HIV-associated neuropathy which is also referred to as HIV-distal symmetrical polyneuropathy (HIV-DSP) [4]. Human immunodeficiency virus-distal symmetrical polyneuropathy in patients can be attributed to the effects of post-anti-retroviral therapy (ART) or the chronicity and debilitation of the condition itself [1,4]. There have been several studies exploring other neurological phenomena associated with HIV such as autonomic neuropathy, peripheral facial paralysis (PFP), and neurocognitive difficulties [5-7]. It is also valuable to learn that there have been various studies that seek to establish the underlying causative mechanism of HIV-related neuropathy. One such study by Rodo et al. was carried out to observe the correlation between mitochondrial deoxyribonucleic acid (mtDNA) deletions and HIV-related neuropathy. It was a retrospective cohort study done on 67 patients and measured deletion burden and neuropathy [8]. The HIV-sensory neuropathy (HIV-SN) can sometimes be disabling as it interferes with daily functioning and has resulted in decreased quality of life among PLHIV [9]. 

The objective of this article is to establish the underlying pathological mechanisms of HIV-SN and highlight current treatment and management strategies. This article also gives an overview of HIV-SN via the comparison of its different existing hypothetical postulations. It's an attempt to delve deeper into the health issue as it will be very useful for PLHIV and would serve as an educational tool for people who are newly diagnosed with HIV and are about to start HAART.

## Review

Pathogenesis

The mechanism of HIV-associated neurological dysfunction is not clearly understood. The pathogenic mechanism behind HIV-DSP can be broadly classified into two distinct hypotheses i.e., the HIV infection itself or, the use of cART.

Neuropathy due to HIV infection

Recent studies have implicated several processes through which a long-standing HIV infection can result in damage to peripheral nerves. It has been theorized that HIV may actively replicate within neurons and cause disruption of the normal physiology of the nerves via a sustained inflammatory environment. To dive deeper into this theory, it is imperative to understand how HIV-1 (a subtype) uses the central nervous system (CNS) as a portal to hide within the neural tissue of the body. According to Krammer-Hammerle et al., even cART cannot reduce viral replication when HIV-1 enters the CNS via the Trojan Horse hypothesis. The Trojan Horse hypothesis states that HIV-1 enters the CNS via the BBB (blood-brain barrier) and binds to various receptors on the macrophage/monocyte cells of the reticuloendothelial system [10]. Prolonged replication within the monocytes (microglia) causes neuroglial cells particularly astrocytes to release inflammatory cytokines namely chemokine CC motif ligand 2 (CCL2) which promotes the diapedesis of even more inflammatory cells and hence, neurological damage [11,12].

**Figure 1:**
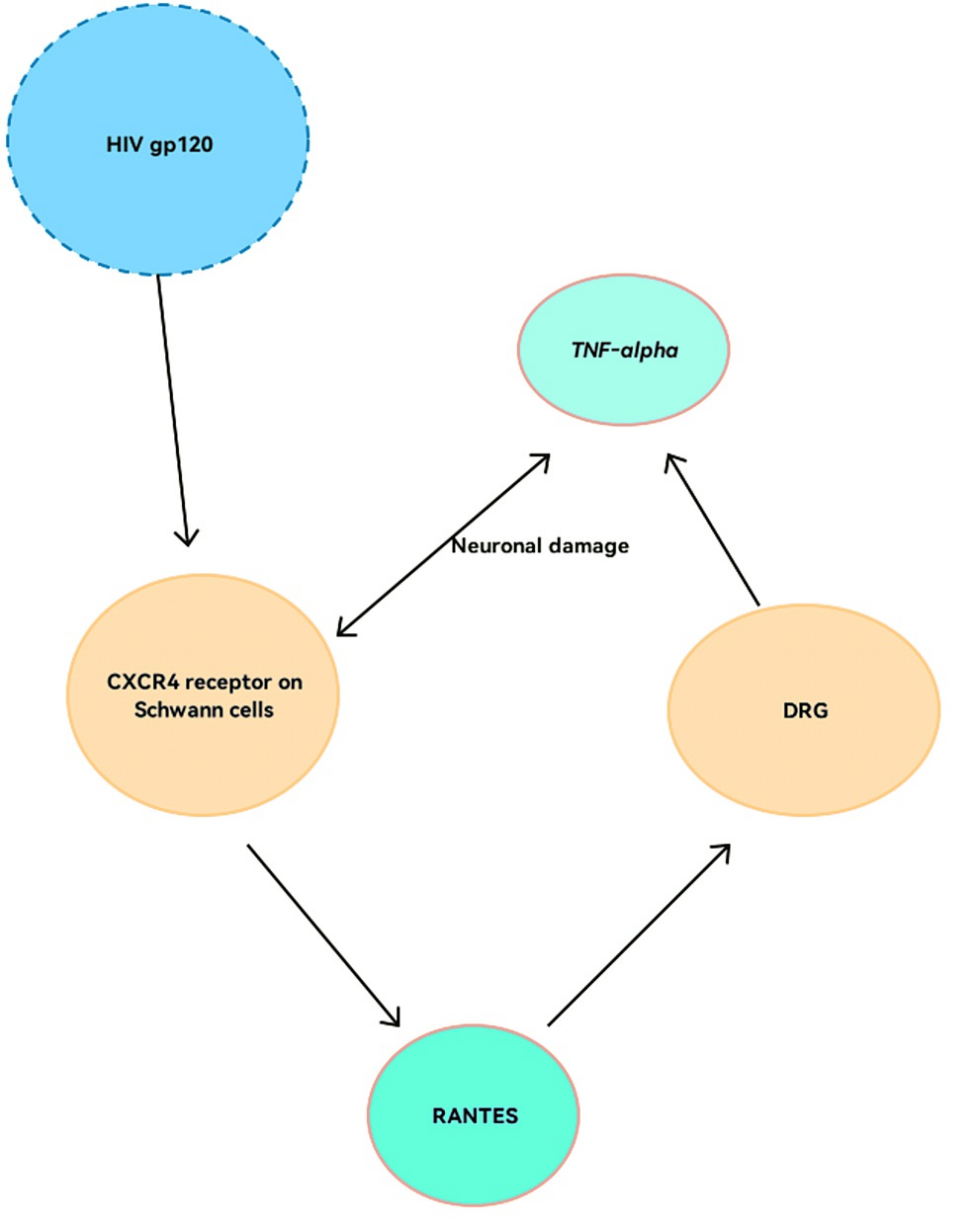
HIV-gp120 protein-induced demyelination via RANTES HIV gp120: Human immunodeficiency virus glycoprotein 120, TNF-alpha: Tumor necrosis factor-alpha, CCR5: C-C chemokine receptor 5, DRG: Dorsal root ganglion, RANTES: Regulated on activates normal T cell expressed and secreted Image created by author Lakshya Motwani

The neurotoxic effects of HIV in the peripheral nerves can be distinctly either direct (viral proteins) or indirect (immune activation), however, it is understood that HIV-DSP is a result of the interplay between the two aforementioned pathways. The HIV-DSP is demonstrated by the glycoprotein 120 (gp120) protein, an envelope antigen of the virion. There has been biological and clinical evidence regarding the neurotoxic effects of gp120 as it causes complement-mediated lysis [13] of the neurons. The gp120 protein activates Schwann cells by binding to the C-X-C chemokine receptor type 4 (CXCR4) receptor and causes them to produce regulated upon activation, normal T cell expressed and presumably secreted (RANTES) also simply known as chemokine ligand 5 (CCL5) that causes the subsequent release of tumor necrosis factor-alpha (TNF-alpha) which will lead to neuronal apoptosis and damage in an autocrine fashion [14,15]. The above fact is further supported by a study done by Jones et al. where they presented that the neuropathic effects of HIV were observed in a culture of a novel dorsal root ganglion (DRG) from transgenic rats that expressed human macrophages infected with HIV containing the CD4 as well as CCR5 receptors, and Schwann cells, with the result of ongoing neuronal demyelination and programmed cell death (Figure 1) [16]. The dendrites or "neurites of these neurons are the most susceptible to damage leading to 'bald' neuronal bodies" [17]. Thus, HIV infection causes direct as well as indirect neurotoxic effects which leads to long-term debilitation and manifests HIV-DSP.

HIV-sensory neuropathy due to HAART

The HIV-SN and other neurological sequelae are also determined by the genetic susceptibility of the patient which is explained by genetic polymorphisms (GP) and single nucleotide polymorphisms (SNP) in the mitochondrion as a result of large deletions [8,18]. These changes in the mitochondria are also associated with increased disease susceptibility in different demographics, as explained by the differences in mitochondrial bioenergetics and genes [19]. Mitochondrial DNA (mtDNA) is susceptible to mutations as it is independent of the nuclear genome. Mutations of mtDNA have been implicated as the inciting event in the idiosyncratic neuropathy induced by ART [20]. One particular SNP, called calcium/calmodulin-dependent protein kinase kinase 2 (CAMKK2) has been associated with a higher risk of developing HIV-SN in South-African HIV patients [21,22]. In the study by Goullee et al., several haplotypes and SNP were identified in patients with HIV in black South-African patients and the gene CAMKK2 was exhibited as a significant predictor of HIV-SN susceptibility in patients independent of stavudine exposure [21]. To further corroborate this theory, a study by Apostoltova et al. showed a complex interplay between HAART, particularly the NRTI and the mitochondria, that results in HIV-SN. The NRTI and other HAART such as non-nucleoside reverse transcriptase inhibitors (NNRTI) and protease inhibitors (PI) have a complex pathological effect on the DNA polymerase-gamma (DNA pol-gamma) which has been linked to increased neurological dysfunction [23,24]. A study by Ciccosanti et al. devised a novel biomarker for mitochondrial dysfunction in cART-induced HIV-SN known as prohibitin. Samples were taken from ART-naive and ART-exposed individuals, and proteomic analysis revealed that they exhibited lower levels of mitochondrial enzyme and chaperone proteins, in particular prohibitin, in ART-exposed patients compared to ART-naive patients. This study proved the NNRTI-induced neurotoxicity, especially with the use of zidovudine and stavudine [25]. Mitochondrial dysfunction is the link between HIV-SN and lifelong ART therapy. The pathological manifestations of HIV-DSP were measured on nerves with intraepidermal nerve density fiber (IENFD) studies by Lakritz et al. by observing the microscopic changes in simian immunodeficiency virus (SIV) infected DRG that demonstrated monocyte trafficking and proposed chronic inflammation as the resultant decrease in IENFD (Table1) [26].

**Table 1:** Studies demonstrating the pathogenesis of HIV-induced neurotoxicity HIV: Human immunodeficiency virus, SNP: Single nucleotide polymorphism, HIV-SN: HIV sensory neuropathy, DNA: Deoxyribonucleic acid, ART: Antiretroviral therapy, MALDI-TOF: Mass spectrometry-Matrix-assisted laser desorption/absorption ionized-time of flight mass spectrometry, SIV: Simian immunodeficiency virus, IENFD: Intraepidermal nerve fiber density, BrdU-5: Bromo deoxyuridine, MAC387: Antimacrophage antibody clone, DRG: Dorsal root ganglion, CAMKK2: Calcium/calmodulin-dependent protein kinase kinase 2, CCR5: CC chemokine receptor 5, CXCR4: C-X-C chemokine receptor type 4, CD68: Cluster of differentiation 68, CD163: Cluster of differentiation 163

Reference	Study subject	Results	Conclusion
Jones et al. [16]	Transgenic rats containing human microglia, Schwann cells, and nerve fibers were infected with HIV and their changes were observed by using immunophenotyping.	C2V3 virus strain was isolated using sequential analysis from peroneal nerve fibers that exhibited dual tropic HIV (CCR5 and CXCR4) causing neurological damage primarily via macrophages.	HIV causes macrophage-induced neuronal damage
Goulee et al. [21]	153 HIV-positive black South African patients exposed to stavudine	45 SNPs were isolated using Taqman fluorescent probes in the DNA samples of patients and four were implicated in the pathogenesis of HIV-SN. The haplotypes were derived using fastPHASE algorithm and logistic regression analysis was applied using appropriate demographic factors (excluding patients that carried CAMKK2)	CAMKK2 has the highest association with HIV-SN with two SNPs and six haplotypes predicting the status of HIV-SN in South African patients
Ciccosanti et al. [25]	ART-exposed as well as ART-naive HIV patients	Mitochondrial proteome isolated from HIV-infected patients via gel electrophoresis	Results via MALDI-TOF mass spectrometry revealed that mitochondrial chaperone proteins and cytoskeletal elements get destroyed in HIV-infected patients. The use of ART further enhanced this effect.
Lakritz et al. [26]	16 SIV-infected CD8-infected lymphocyte rhesus macaque model (Macaca mullata) out of which four were control and the rest 12 infected with SIV.	An increase in CD68(+) and CD163(+) macrophages in DRGs. MAC387(+) recruited macrophages were increased, along with BrdU(+) cells. 78.1% of all BrdU(+) cells in DRGs were also MAC387(+)	IENFD decreases consistently with HIV infection and (MAC387+)(BrdU+) macrophages were recruited for significant neuronal inflammation

Literature review of the clinical implications of HIV neuropathy

Neurological complications of HIV such as HIV-DSP, peripheral sensory neuropathy, and autonomic neuropathy were considered a constellation of conditions in the pre-ART era. There were no reported cases at the beginning of the HIV epidemic. However, the autopsies of patients that died due to HIV showed evidence of neuropathy in nearly all the cases. It is also interesting to note that in the post-ART era, the neurological signs and symptoms of PLHIV manifested and were present in about 50% of individuals [27]. Of these individuals, 57% suffer from HIV-DSP and 38% from neuropathic pain [27]. The most common form of peripheral neuropathy in HIV patients is HIV-DSP [28]. It's also observed that nearly two-thirds of the individuals having HIV-SN may or may not encounter symptoms until the neurological dysfunction can be prevented [29]. Paraesthesia is considered the most significant preceding symptom of eventual neuropathic pain. This is elucidated from a longitudinal prospective cohort study done on 267 PLHIV by Diaz et al. in the central nervous system HIV antiretroviral therapy research (CHARTER) study of which 21% were female, with a mean age of 56 years, and were suffering from paraesthesia and pain. The outline of this study was to predict the outcome of neuropathic pain in people who suffered from paraesthesia which was significant among nearly 23% of 100 patients on follow-up after 12 years (odds ratio (OR) 1.56; 95% confidence interval (CI) 1.18, 2.07) [30].

Age is considered a risk factor for developing HIV-DSP. A study by Sakabumi et al. done on 3379 ambulatory adults suffering from HIV in 2019, identified that about 52% suffered from chronic distal sensory polyneuropathy (cDSP) and reported that HIV+ individuals suffered from balance issues more than HIV- with statistical significance (p=0.001) [31]. It was also noted that older individuals suffered from cDSP more than younger ones [31]. 

In recent years, with the introduction of HAART, the overall quality of life of HIV patients has improved. The disease progression and mortality have been curbed immensely due to HAART. However, as discussed in the earlier sections, cART further increases the risk of HIV-SN both by direct effects and by increasing the longevity of HIV patients. 

A prevalence study conducted by Mullin et al. on 326 HIV-infected adults both male and female in Tanzania, was carried out to study the risk of HAART, namely stavudine and didanosine, in the occurrence of HIV-SN. They were divided into four groups based on whether or not they received ART and the CD4 count being less than or more than 200 cells/cu mm [32].

The HIV-DSP was determined by at least one symptom of reduced ankle reflexes, reduced vibration, or both. In addition, a set of standardized questions asked participants whether they suffered from any motor impairment in hands, arms, legs, and feet as well as if they experienced any occupational impairment. The questionnaire contained questions in regards to whether the participant had difficulty buttoning a shirt, opening a tight jar, tying shoelaces (hands); combing their hair (arms); climbing up the stairs, standing from a seated position (legs), gait unsteadiness, and operating car pedals (feet). 

Nearly 80 patients were present in each of the four groups, namely the ART-naive group with a CD4 count of more than or less than 200 cells/cu mm and the ART exposed group with a CD4 count of more than or less than 200 cells/cu mm. The time exposed to cART in the group 'no ART/CD4<200' was an average of 12 months, and an average time of 18 months in the group containing patients with 'ART/CD4<200'.

It was found that numbness was the most common symptom, and HIV-DSP was present in 43% of patients with ART/CD4<200 and 20% in patients with no ART/CD4>200. Male (adjusted odds ratio (aOR) 1.9, 95% CI 1.2-3.3) and older individuals (aOR 2.7, 95% CI 1.1-6.2 for age 40 + vs. <30 years) had a higher prevalence of HIV-DSP. It was concluded that the use of ART posed an additional risk for HIV-DSP, however, it exists among individuals not taking ART as well [32].

In comparison to the use of ART such as zidovudine (d4T) and didanosine (ddi) increasing the risk for HIV-SN, the prevalence of HIV-SN has remained stable in ambulatory patients even after the cessation of the use of nucleoside reverse transcriptase inhibitors (NRTI) such as d4t and ddi. This was attributed to the increased exposure to indinavir, another component of HAART that reduces viral protein maturation [33].

Furthermore, according to a study conducted by Childs et al., the risk of developing HIV-related neuropathy is higher in individuals with a higher HIV RNA load. In the study, 1,604 men with a seropositive status were followed up for 10 years, and 213 with HIV-SN and HIV-associated dementia (HAD) were identified. Their HIV RNA and CD4 levels were measured and used as predictors for the neurological outcomes. An HIV RNA >30,000 copies/mL had a relative risk for HIV-associated neurocognitive dysfunction 8.5 times (p < 0.001) than that of those with <3,000 copies/mL, and those with CD4 counts <200 cells/cu mm had a 3.5-fold (p=0.003) greater hazard relative to those with CD4 counts >500 cells/cu mm (Table 2) [34].

**Table 2:** Epidemiological studies that investigate the predisposing risk factors for the development of HIV-SN PLHIV: People Living with HIV, CHARTER: Central nervous system HIV antiretroviral therapy effects research, cART: Combined antiretroviral therapy, HIV-RNA: Human immunodeficiency virus ribonucleic acid, NRTI: Nucleoside reverse transcriptase inhibitors, MAC: Mycobacterium avium complex, HIV-SN: HIV sensory neuropathy, HAD: HIV-associated dementia, HIV-DSP: HIV-distal symmetrical polyneuropathy, OR: Odds ratio, aOR: Adjusted odds ratio, CI: Confidence interval

Reference	Study design	Methods	Results	Conclusion
Diaz et al. [30] (2021)	Longitudinal Prospective Cohort Study.	265 PLHIV in CHARTER study with baseline evaluation and follow-up for 12 years. 100 patients with the mean age of the patients being: 56 +/- 8 years. 21% of the patients were female. Nearly all were on cART and 82% suppressed HIV-RNA levels (<50 copies/ml)	Increased risk of pain from follow-up (OR 1.56; 95% CI 1.18, 2.07) was significant, however, paraesthesia predicted a higher risk on follow-up (OR 1.96, 95% CI 1.51, 2.58).	Paraesthesia is an important predictor of pain on follow-up in HIV-SN patients
Sakabumi et al. [31] (2019)		3379 PLHIV were prospectively studied and followed up for 10 years for the development of balance issues. History of balance-related problems was taken and coded as minimal-to-none or mild-to-moderate.	52% met the criteria for HIV-DSP and 11% developed balance issues which were common among patients with HIV-DSP (OR 3.3 (2.6-4.3), p=0.001)	Balance issues are more pronounced in patients with HIV-DSP
Mullin et al. [32] (2011)	Prevalence study	326 PLHIV were divided based on ART exposure and CD4 count less than or more than 200 cells/cu mm	Numbness was the most common symptom and HIV-DSP was present in 43% of all patients. Male aOR 1.9, 95% CI 1.2-3.3) and older individuals (aOR 2.7, 95% CI 1.1-6.2 for age 40 + vs. <30 years) had a higher prevalence of HIV-DSP.	The use of ART posed an additional risk for HIV-DSP, however, it exists among individuals not taking ART as well.
Smyth et al. [33] (2007)	A cross-sectional comparative study using convenience sampling	100 patients attending the clinic over two weeks were compared to patients that attended the clinic between 1993 and 2001.	HIV-SN remains much more common now (42%, p<0.0001) as compared to in 2001 (13%, p<0.0001)	HIV-SN remained common among ambulatory patients in 2006 due to increased patient age and exposure to NRTI as compared to 1993 when it was due to MAC infection and in 2003 where it was independent of NRTI use
Childs et al. [34] (1999)		1604 seropositive men were followed up for 10 years for HIV-SN and HAD. Their CD4 and HIV-RNA levels were recorded accordingly.	77 patients with HAD and 213 patients with HIV-SN were identified. Baseline levels of HIV-RNA more than 3000 copies/ml and CD4 count below 500 cells/cu mm were predictive of both neurological outcomes.	There was twice the risk of HIV-SN in male patients having >10,000 copies/ml and CD4 <750 cells/cu mm as compared to those with <500 copies/ml and CD4 >750 cell/cu mm. High levels of HIV-RNA levels may be the initiating event in the neurological complications of HIV such as HIV-SN and HAD.

It can be concluded that the modifiable risk factors for HIV-SN are HIV-RNA load, CD4 count, and exposure to cART and the non-modifiable risk factor are prolonged survival of the condition and old age. Other causes of peripheral neuropathy such as diabetes mellitus (DM) and the use of chemotherapeutic drugs are considered. Low hemoglobin has also been proposed as a risk factor for aggravating neuropathic pain in HIV patients [35].

Diagnosis

There is a wide spectrum of symptoms in HIV-peripheral neuropathy (HIV-PN) that include pain, hypoaesthesia, weakness, or a complete lack of symptoms altogether. They usually appear in a glove-and-stocking distribution of the extremities. These symptoms can be associated with burning pain or can be painless [36]. A detailed clinical examination is necessary to demonstrate these manifestations. They may appear in glove-and-stocking distribution of the extremities and the bedside techniques will show absent or decreased knee reflex, decreased perception of vibration, pinprick, or temperature [1]. Neuropathy due to HIV or due to cART usually has significant overlap with presentation and cannot be differentiated [37]. 

The brief peripheral neuropathy score (BPNS) and the total neuropathy score are used frequently to assess the extent and severity of the neuropathy [38,39]. The BPNS is especially a quick assessment tool and when compared with TNS, its sensitivity and specificity are 35% to 49% and 88% to 90% respectively, with a positive predictive value (PPV) of 72%. It is considered highly useful and an easy bedside method to diagnose HIV-SN [40]. It can also be accessed in resource-poor regions and can effectively diagnose the condition. According to Cherry et al., numbness as a symptom had the greatest diagnostic value for an assessment of HIV-PN [40,41].

In 2016, another study attempted to devise a neuropathy assessment tool specific for HIV. Woldeamanuel et al. devised the clinical HIV-associated neuropathy tool (CHANT). The CHANT is a four-item clinical tool that checks for signs such as ankle reflexes and vibration sense and symptoms such as pain and paraesthesia. It was used on patients in the UK and South Africa that consisted of two cohorts: patients with pain and without pain. The patients with pain were assessed by douleur neuropathique en 4 questions (DN4) and the patients with only numbness were assessed by Utah early neuropathic score (UENS). The sensitivity of CHANT in patients was computed to be 100% in London, the UK whereas, in patients in Johannesburg, South Africa it was 74.4%. The specificity of the CHANT score remained to be 85% in both regions [42].

There is no established confirmatory diagnostic test for HIV-DSP. The diagnosis of HIV-SN is clinical and may be confirmed with electrodiagnostic tests such as nerve conduction studies (NCS), nerve biopsies, etc. Not only do they help in detecting the presence of neuropathy but they can also help in determining the severity, extent, and progression of the disease [43].

Electrodiagnostic testing can be used as a confirmatory diagnostic tool for HIV-SN. A study was conducted to measure HIV-SN with quantitative sensory testing (QST)and compared it with electrodiagnostic parameters with painless or painful neuropathy. Forty patients with neuropathy related to HIV were studied out of which 15 had painful peripheral neuropathy (PPN) and the remaining 25 did not. The QST measurements were acquired from the visual analog scale (VAS) in the form of suprathermal stimuli and thermal threshold. These values were compared to NCS taken from the patients [44].

In contrast, less invasive methods such as stimulated skin wrinkling (SSW), skin vasoconstrictive response (SVCR), and sudoscan have also been used for electrodiagnostic testing. Sudoscan is a simple doppler probe that detects the interaction between the sweat chloride and the ultrasound probe for small nerve fibre studies. The SSW detects the presence of undulations or wrinkles on the skin after submersion in a eutectic mixture of local anesthetic (EMLA) that has a sensitivity of 81.3% and specificity of 67.0% [45].

Though there is no proven gold standard method to diagnose neuropathy, a combined approach of high clinical suspicion, examination, and electrodiagnostic tests must be performed on all patients with HIV regardless of the use of HAART [37].

Treatment

The treatment of HIV-SN aims to reduce the disabling neuropathic symptoms such as burning pain, hypoaesthesia, and paraesthesia in order to improve the daily functioning of the individual as well as minimize further neurological damage to prevent further damage. There is no concrete intervention to achieve the aforementioned goals as it is difficult to fully suppress the symptoms [46]. Currently, the realistic goal of palliative care in HIV-SN is to reduce the symptoms by a margin of 30% to 50% [47].

The treatment of neuropathic pain in HIV is similar to that of DM-associated neuropathy and drug-induced neuropathy as it aims to provide symptomatic relief to patients. It features antidepressants that include tricyclic antidepressants (TCA) such as amitriptyline, opioid analgesics, and anti-epileptic drugs (AED) such as lamotrigine (LTG), gabapentin, and pregabalin [48]. These therapeutic regimens are primarily made for diabetic neuropathy and post-herpetic neuralgia, however, they have demonstrated results superior to those of placebo in patients with HIV-SN [49]. The use of carbamazepine is avoided as its primary side effect of myelosuppression worsens the progression of AIDS in patients [50]. Other therapies include topical capsaicin patches, non-steroidal anti-inflammatory drugs (NSAIDs), and selective serotonin reuptake inhibitors (SSRI) such as duloxetine [37]. Although several agents are being studied for the treatment of HIV-SN, there is no single drug approved by the United States Food and Drug Administration (FDA) yet [50].

Discontinuation of cART is imperative to patients with progressively increasing signs of HIV-DSP, as they have been implicated to be neurotoxic. Cessation of HAART is important at least four to six weeks before initiating therapy for HIV-SN. Sometimes it may take up to 16 weeks and the symptoms might worsen in the middle of this period [51,52].

Discussion of various antiepileptic medications and their comparisons show that they all exhibit varying efficacies and are still under trial for the time being. A randomized clinical trial studying the effects of LTG and contrasted with placebo was carried out in 2003 by Simpson et al. that showed a greater reduction in pain and increased tolerance to LTG as compared to placebo significantly (p=0.004 for patients receiving ART and p </=0.02 for patients not exposed to ART) [53]. Pregabalin (an AED) has been studied in detail regarding its efficacy in the treatment of HIV-associated neuropathy. In a randomized, double-blind, placebo-controlled, multinational study conducted in 2014, pregabalin did not show superior efficacy in the treatment of HIV-SN as compared to placebo [54]. Though most of these medications show similar efficacy in the treatment of neuropathy, there have to be special considerations for neuropathy in conditions such as HIV-SN, trigeminal neuralgia (TN), and chronic radiculopathy [55].

Gabapentin (GBP), another antiepileptic medication, has recently been evaluated for the treatment of HIV-SN and has subsequently shown efficacy in various controlled clinical studies [56,57]. A multicentric, prospective, double-blind, placebo-controlled trial containing 26 patients of which 15 received GBP and 11 received placebo were conducted by Hahn et al. in 2004. The results showed that GBP had superior efficacy (calculated on the VAS) as compared to placebo in reducing neuropathic pain and sleep interference [56]. Gabapentin is not protein-bound, making its side effect profile more tolerable in comparison to other AEDs [57]. However, its use as a mainstay for treatment is unclear due to the small sample size of these trials about HIV-SN alone [58].

The TCAs have also been considered a potential treatment for HIV-SN and have been studied in various clinical trials recently. Although TCAs such as amitriptyline and mexiletine have been used for neuropathic pain, their efficacy in HIV-associated neuropathy remains unknown. Tricyclic antidepressants have proved no benefit in the treatment of HIV-SN, as monotherapy or in conjunction with other non-pharmacological approaches such as acupuncture [59,60]. A study conducting indirect and direct meta-analyses of randomized clinical trials to compare the efficacy between GBP and TCAs has also shown TCAs to be slightly more effective in neuropathic pain relief. However, these studies mention treatment for neuropathic pain associated with diabetes and post herpetic neuralgia, conditions distinct from HIV-SN [61].

Capsaicin is a pungent substance derived from chili peppers that bind to nociceptive fibers via the vanilloid (V1) receptors and alleviates pain. Topical capsaicin patches have been recently targeted for being an exceptional component for the topical treatment of HIV-DSP. In a placebo-controlled, multicentric, randomized trial conducted in 2008, NGX-4010 (a capsaicin derivative) patch was compared to control in 307 participants. It was significant in reducing pain symptoms over a course of 12 weeks (22.8%) as compared to the control (10.7%) (p=0.0026). The mean pain reduction calculated numerically from a numeric pain rating scale (NRPS) for NGX-4010 at the 30, 60, and 90-minute marks were 27.7%, 15.9%, and 24.7%, respectively [62].

Contrary to the aforementioned study of capsaicin, topical lidocaine gel has been studied for the use of HIV-DSP but with unsatisfactory results, as it remained ineffective in maintaining control over the symptoms and can only be considered as adjunct therapy [63].

The WHO analgesic ladder is an effective guideline for pain management in patients with HIV-SN, even though it is for patients with chemotherapy-related pain. It starts with over-the-counter pain medications such as NSAIDs and acetaminophen for mild pain and proceeds to opioid analgesics for moderate and severe pain [64].

For severe pain, opioid analgesics have been used frequently as they are highly effective in controlling the patient’s symptoms and reducing pain-related emergencies. Maintenance therapy with opioids is not preferred due to its dependence potential and increased risk of relapse in previous addicts [56]. Neuroprotection is also a prospective theory that can not only give palliative relief but also promote nerve regeneration. Acetyl-carnitine agents have shown promising results in reducing the progression of this disease [65,66]. Neurotropic factors may also contribute to nerve health and regeneration in HIV-induced neurotoxicity [67].

To conclude, it is important to maintain CD4 levels above 500 cells/cu mm and discontinue cART therapy if possible to prevent HIV-SN.

## Conclusions

Thus, we have reviewed the possible theories that support that HIV-associated neuropathy is a growing concern with the advent of cART and the prolonged survival of HIV-infected individuals. Recent studies have thrown more light on the condition and its evolution in people who suffer from HIV. In this article, we have delineated the exact source of the neurological dysfunction faced by HIV patients in the form of HIV-DSP by reviewing the various pathological mechanisms that are involved. An emphasis is placed on the disease statuses in the individual's pre and post-cART era to better understand the neurotoxic effects of these medications and are compared to the overarching effects of HIV alone. These differences will help the medical community discover a novel approach to preventing neuropathy in HIV. Studies of the various trends in the implied underlying causes of neuropathy date back to the beginning of the HIV epidemic, such as the opportunistic infections specific to it and the overall disease progression characterized by the CD4 count. The same is stated in this article as well. Apart from the prevalence of the condition, various prognostic factors such as paraesthesia have been studied in order to improve our radar of the disease in patients with HIV. The current approach to HIV-SN and its overlapping features with other causes of peripheral neuropathy demonstrated that a clinical examination is the most important clue for a healthcare professional to suspect the disease. It is crucial to highlight this complication of HIV/AIDS as it presents itself silently and may cause severe dysfunction in an individual already battling lifelong problems.

Even though nearly half of HIV-infected persons suffer from this condition, there is no single diagnostic criteria or management approach for HIV-SN. In light of this disability and prevalence, it is of utmost importance to delve deeper into the issue and find a solution promptly. With increased encounters with this entity, recent studies have been developed to find a more targeted approach to HIV-SN to minimize the long-term and irreversible neurological sequelae by the use of neurotrophic factors, supplements like acetylcarnitine, and preventive efforts to reduce its prevalence. 
